# Characterization of fluvoxamine degradation products in postmortem blood by liquid chromatography coupled with quadrupole-Orbitrap mass spectrometry

**DOI:** 10.1007/s11419-026-00763-6

**Published:** 2026-03-16

**Authors:** Yoshikazu Yamagishi, Kazuaki Takahashi, Hiroyuki Inoue, Sayaka Nagasawa, Hirotaro Iwase, Yasumitsu Ogra

**Affiliations:** 1 Department of Legal Medicine, Graduate School of Medicine, Chiba University, 1-8-1 Inohana, Chuo, 260-8670 Chiba Japan; 2 Graduate School of Horticulture, Chiba University, 1-33 Yayoi-cho, Inage, 263-8522 Chiba Japan; 3 Laboratory of Forensic Toxicology, Graduate School of Pharmaceutical Sciences, Chiba University, 1-8-1 Inohana, Chuo, 260-8675 Chiba Japan; 4 Laboratory of Toxicology and Environmental Health, Graduate School of Pharmaceutical Sciences, Chiba University, 1-8-1 Inohana, Chuo, 260- 8675 Chiba Japan

**Keywords:** Fluvoxamine, Formylation, Postmortem product, Hemoglobin, LC-Q-Orbitrap-MS

## Abstract

**Purpose:**

Fluvoxamine (FLV) is a selective serotonin reuptake inhibitor primarily used to treat obsessive-compulsive disorder. In Japan, FLV is linked to lethal intoxication and suicide cases. Therefore, it is important to establish its exact concentration in postmortem (PM) blood. It has been reported that blood FLV concentration decreases over time. In this study, we aimed to clarify the mechanisms underlying changes in FLV concentration in human blood over time.

**Methods:**

We examined whether FLV underwent hydroxylation and/or oxidation in a Fenton reaction mixture containing FeCl_2_, hemoglobin (Hb), or hydrogen peroxide (H_2_O_2_) after incubating at 37 °C for 24 h. In addition, we measured FLV and its degradation products in human blood by liquid chromatography coupled with quadrupole-Orbitrap mass spectrometry.

**Results:**

Mass spectrometric result indicated the formation of (*E*)-*N*-(2-(((5-methoxy-1-(4(trifluoromethyl)phenyl)pentylidene)amino)oxy)ethyl)formamide (FLV-CHO), (*E*)-5-hydroxy-1-(4-(trifluoromethyl)phenyl)pentan-1-one-*O*-(2-aminoethyl)oxime (FLD), and (*E*)-5-(hydroxymethoxy)-1-(4-(trifluoromethyl)phenyl)pentan-1-one-*O*-(2-aminoethyl)-oxime (FLV-OH) in the reaction mixture. In addition, FLV-CHO concentration in human blood was strongly correlated with percentage FLV degradation relative to its initial concentration.

**Conclusions:**

Our results indicate that FLV-CHO, formed by Hb/H_2_O_2_-mediated FLV degradation in blood, is a potential biomarker for correcting FLV concentration in PM blood.

**Supplementary Information:**

The online version contains supplementary material available at 10.1007/s11419-026-00763-6.

## Introduction

Forensic toxicology is an applied field of toxicology essential for safeguarding people’s rights. Forensic toxicologists (FTs) analyze biological samples, such as blood, urine, and tissues, obtained during autopsy to provide concrete evidence for the justice system. In particular, accurately determining blood drug concentration at the time of death is crucial to confirming death by drug intoxication. However, FTs have been baffled because blood drug concentration in persons who have died from drug intoxication is much lower at autopsy than at the time of death. The concentration of drug in blood at autopsy may not accurately reflect that at the time of death [1].

Fluvoxamine (hereinafter “FLV”), 2-[(*E*)-[5-methoxy-1-[4-(trifluoromethyl)phenyl]pentylidene]amino]oxyethanamine, is an oxime *O*-ether that is benzene substituted by a (1*E*)-*N*-(2-aminoethoxy)-5-methoxypentanimidoyl group at position 1 and a trifluoromethyl group at position 4. As a selective serotonin reuptake inhibitor (SSRI), it is primarily used to treat obsessive-compulsive disorder (OCD) [2]. It works by increasing the levels of serotonin, a neurotransmitter, in the brain, thereby improving mood and reducing anxiety and obsessive thoughts. Unlike other SSRIs, it is primarily used for OCD, although it is also prescribed for depression and other anxiety disorders. Common side effects include nausea, drowsiness, and insomnia. It is important to take it as prescribed and avoid stopping abruptly, as this can lead to withdrawal symptoms. As with all antidepressants, it may take several weeks to feel its full effects. Patients should be monitored for potential side effects and behavioral changes, especially at the beginning of treatment. FLV is known to be one of the causes of death by suicide or accidental intoxication in Japan [3]. The lethal blood concentration of FLV is > 2800 ng/mL [4]. It has been reported that FLV concentration in blood decreases in a time-dependent manner [5]. In Japan, a dead body is routinely kept at 4 °C for a number of days before autopsy [6]. Because of this, blood FLV concentration in persons who have died from intoxication is much lower than that at the time of death. Currently, the reasons for the observed changes in blood FLV concentration are poorly understood.

Hemoglobin (Hb) is an important contributor to the decrease in drug and pesticide concentrations in blood after death [7–13]. Hb is released from erythrocytes by hemolysis following death [14]. On the other hand, hydrogen peroxide (H_2_O_2_) is generated by the dismutation of O_2_^•–^ from HbO_2_ autoxidation [15]. The Fenton reaction is induced by H_2_O_2_ and Hb in blood [11]. Recent studies have demonstrated the postmortem (PM) degradation of drugs, such as amlodipine, bromazepam, etizolam, paliperidone, and zolpidem, by Hb/H_2_O_2_ [7–11]. As far as we know, there are no reports of interactions between FLV and Hb. However, we surmise that Hb may affect changes in blood FLV concentration.

In the present study, we measured FLV and its degradation products in a Fenton reaction mixture and in human blood. To characterize FLV and its degradation products, liquid chromatography coupled with quadrupole-Orbitrap mass spectrometry (LC-Q-Orbitrap-MS) was used. Orbitrap-MS can provide accurate mass data, and tandem MS, such as Q-Orbitrap-MS, can provide structural information [7–10, 16]. Thus, LC-Q-Orbitrap-MS is well-suited for detecting untargeted molecular species, such as unknown FLV degradation products formed in blood.

## Experimental procedures

### Chemicals and Instruments

All solvents were either LC-MS or HPLC grade. Acetonitrile (ACN) and water were purchased from Kanto Chemical Co., Inc. (Tokyo, Japan). FLV (CAS No.: 54739-18-3), FeCl_2_, 100 mM phosphate buffer (pH 7.4), 1 M ammonium formate solution, H_2_O_2_ (30%), and formic acid were obtained from Fujifilm Wako Pure Chemical (Osaka, Japan). Diazepam-*d5* was purchased from Hayashi Pure Chemical Ind., Ltd. (Osaka, Japan). Hb (Human, H7379) was from Sigma-Aldrich (St. Louis, MO, USA). Human blood was purchased from BioIVT (London, UK).

Details of the instrumentation settings are shown in Table S1. LC-Q-Orbitrap-MS was employed to detect FLV and its degradation products in the Fenton reaction mixture. A Vanquish Flex Binary LC system fitted to a Q-Exactive plus Orbitrap mass spectrometer (Thermo Fisher Scientific, Waltham, MA, USA) was employed.

### Recovery of FLV from the Fenton reaction mixture

The reaction conditions were based on our previous reports [7–11]. We used acetonitrile to dissolve FLV in blood. The recovery of FLV from the Fenton reaction mixture was evaluated as follows. A 100 µL aliquot of the reaction mixture containing FLV (1 µg/mL), H_2_O_2_ (0.3 mg/mL), Hb (100 mg/mL), or FeCl_2_ (40 mM) was prepared. Control samples contained FLV in 100 mM phosphate buffer (pH 7.4) without Hb, FeCl_2_, or H_2_O_2_. The reaction mixture was incubated at 37 °C for 24 h before the addition of 1.9 mL of ACN containing diazepam-*d5* (25 ng/mL) as an internal standard (IS). The reaction mixture was sonicated, vortexed, and centrifuged (10,000 ×*g*) for 10 min. The supernatant was analyzed by LC-Q-Orbitrap-MS to determine FLV concentration. The corresponding extracted ion was *m/z* 319.1628 with an *m/z* tolerance of 5 ppm.

### Detection and characterization of unknown FLV degradation products

The reaction and pretreatment conditions were the same as those used for the “Recovery of FLV from the Fenton reaction mixture”. Following the pretreatment described above, the Fenton reaction mixture was analyzed by LC-Q-Orbitrap-MS. Compound Discoverer software (version 3.3; Thermo Fisher Scientific) to extract unknown FLV degradation products from the mass spectra by difference analysis.

### Detection of FLV and FLV degradation products in blood

A 100 µL aliquot of blood containing FLV (1 µg/mL) was prepared and incubated at 37 °C. Sampling times were 0, 24, 96, and 168 h. To this, 400 µL of ACN containing IS (25 ng/mL) was added, and the mixture was vortexed and centrifuged at 10,000 ×*g* for 10 min. The supernatant was subjected to LC-Q-Orbitrap-MS. Extracted ions of FLV, FLV-UK-1, FLV-UK-2, and FLV-UK-3 were detected at *m/z* 319.1628, 347.1577, 305.1471, and 335.1564 with an *m/z* tolerance of 5 ppm, respectively.

### Statistical analyses

Comparisons among four groups were performed using the Tukey test and Dunnett’s multiple comparison test. In the Tukey test, the level of significance was set at *p* < 0.05, and groups with different letters were significantly different. In Dunnett’s multiple comparison test, single and double asterisks (*) indicate levels of significance at *p* < 0.05 and *p* < 0.01, respectively. Data are shown as means ± standard deviation (SD) (*n* = 4).

## Results

### Changes in FLV concentration in the Fenton reaction mixture

**Fig. 1:**
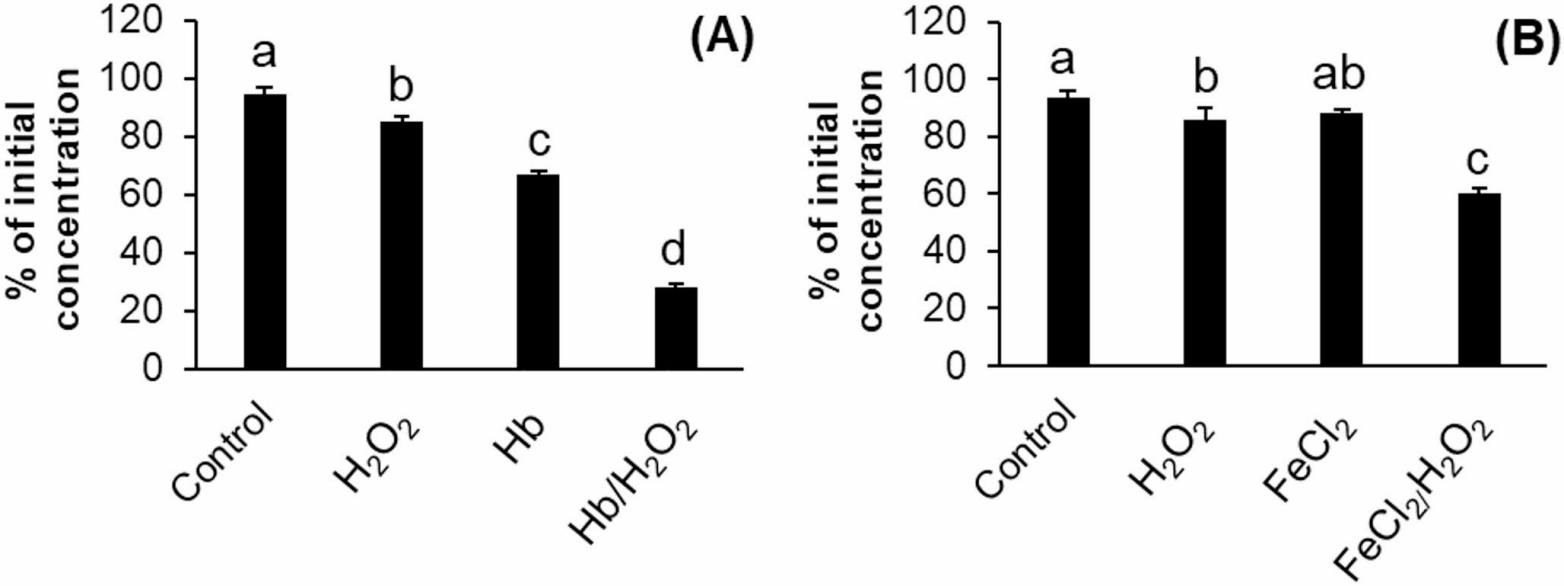
Effects of Fenton reaction reagents on FLV concentration. Fenton reaction mixtures containing 1 µg/mL FLV and H_2_O_2_ (**A** and **B**), Hb (**A**), Hb/H_2_O_2_ (A), FeCl_2_ (**B**), and FeCl_2_/H_2_O_2_ (**B**) were incubated for 0 and 24 h. The control was composed of 0.1 M phosphate buffer (pH 7.4) and FLV. Temperature was maintained at 37 °C. Data are expressed as means ± standard deviation (SD) (*n* = 4). The Tukey test was performed for comparisons among four groups. The level of significance was set at *p* < 0.05, and groups with different letters were significantly different

FLV concentrations in H_2_O_2_, Hb, and Hb/H_2_O_2_ reaction mixtures maintained at 37 °C were decreased after incubation for 24 h compared with control (Fig. 1A). The percentages of undegraded FLV in control and H_2_O_2_, Hb, and Hb/H_2_O_2_ reaction mixtures at 24 h relative to those at 0 h were 94.8 ± 2.2%, 85.2 ± 2.0%, 66.9 ± 1.3%, and 28.3 ± 1.0%, respectively. The decrease in FLV concentration in control and H_2_O_2_, Hb, and Hb/H_2_O_2_ reaction mixtures followed the order Hb/H_2_O_2_ > Hb > H_2_O_2_ > control.

FLV concentrations in H_2_O_2_, FeCl_2_, and FeCl_2_/H_2_O_2_ reaction mixtures maintained at 37 °C were decreased after incubation for 24 h compared with control (Fig. 1B). The percentages of undegraded FLV in control and H_2_O_2_, FeCl_2_, and FeCl_2_/H_2_O_2_ reaction mixtures at 24 h relative to those at 0 h were 93.4 ± 2.5%, 85.6 ± 4.6%, 88.2 ± 0.9%, and 60.1 ± 1.6%, respectively. The decrease in FLV concentration in control and H_2_O_2_, FeCl_2_, and FeCl_2_/H_2_O_2_ reaction mixtures followed the order FeCl_2_/H_2_O_2_ > H_2_O_2_ ≥ FeCl_2_ ≥ control.

### Detection/characterization of unknown FLV degradation products in the Fenton reaction mixture

**Fig. 2:**
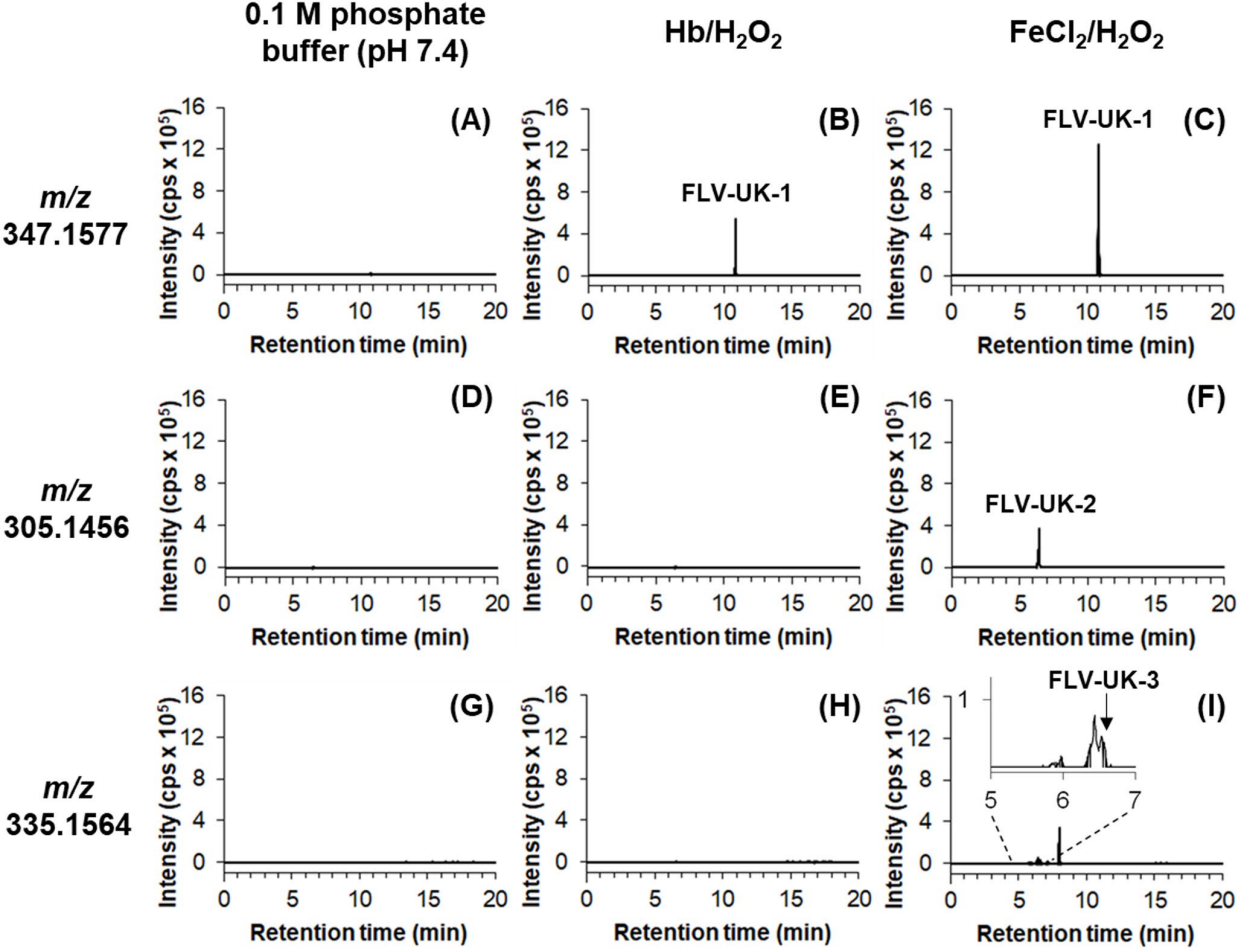
Elution profiles of FLV-UK-1 at *m/z* 347.1577 (**A**–**C**), FLV-UK-2 at *m/z* 305.1456 (**D**–**F**), and FLV-UK-3 at *m/z* 335.1564 (**G**–**I**) in 0.1 M phosphate buffer (pH 7.4) (**A**, **D**, and **G**), Hb/H_2_O_2_ reaction mixture (B, E, and H), and FeCl_2_/H_2_O_2_ reaction mixture (**C**, **F**, and **I**) over 24 h at 37 °C. A reaction mixture containing 1 µg/mL FLV and Hb/H_2_O_2_ or FeCl_2_/H_2_O_2_ was incubated for 24 h at 37 °C. The retention times of FLV-UK-1, FLV-UK-2, and FLV-UK-3 were 10.8, 6.4, and 6.6 min, respectively

Difference analysis of mass data for control and Hb/H_2_O_2_- and FeCl_2_/H_2_O_2_-treated samples at 24 h was performed as described in Experimental Procedures. One ion with *m/z* 347.1577, detected in the Hb/H_2_O_2_-treated sample, was extracted (Fig. 2B). Three ions with *m/z* 347.1577, 305.1458, and 335.1564, detected in the FeCl_2_/H_2_O_2_-treated sample, were extracted (Fig. 2C and F, and 2I). These three unknown degradation products, having retention times of 10.8 min, 6.4 min, and 6.6 min, were designated FLV-UK-1, FLV-UK-2, and FLV-UK-3, respectively. FLV-UK-2 and FLV-3 were not detected in the Hb/H_2_O_2_-treated sample.

**Fig. 3:**
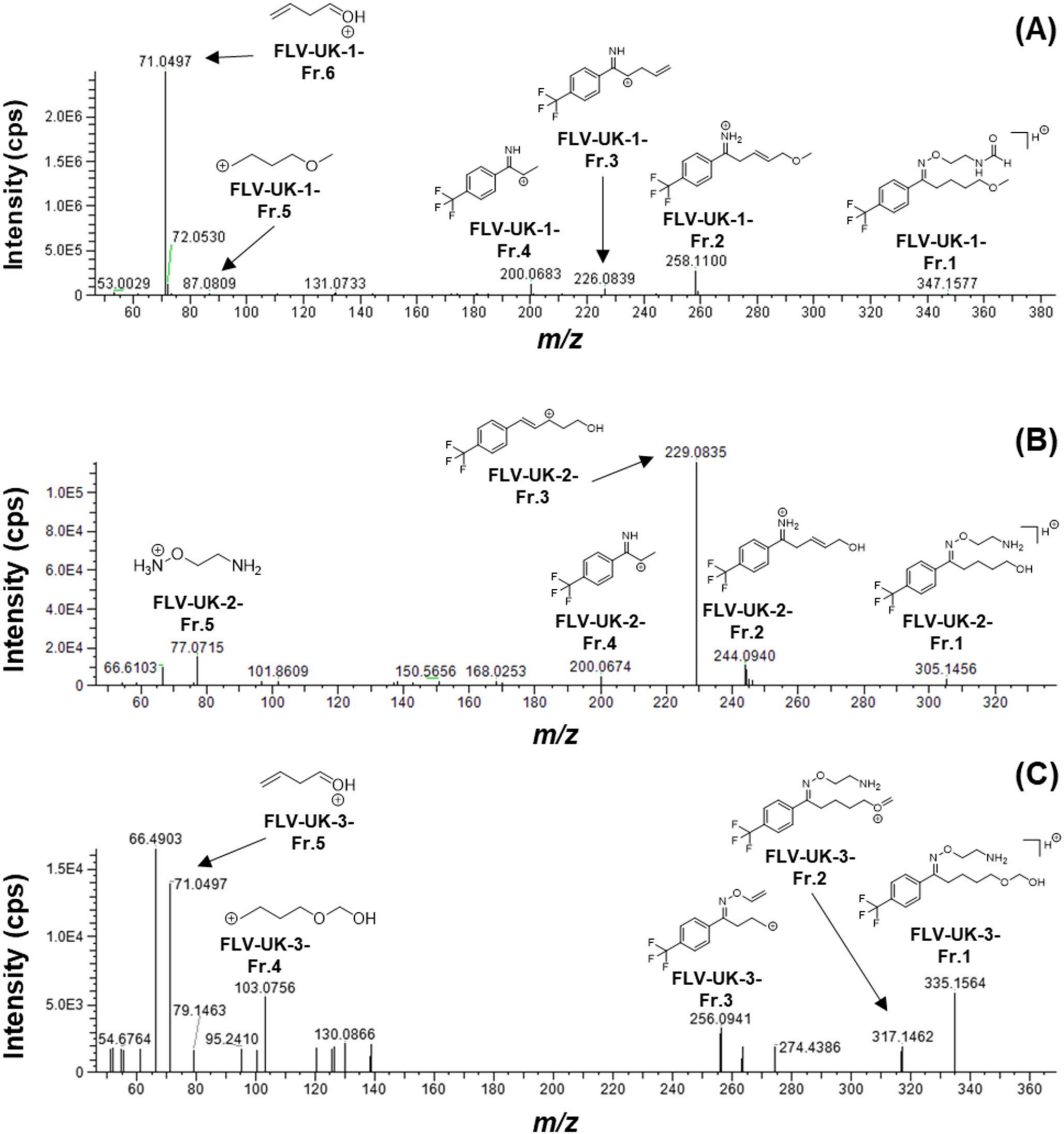
MS/MS spectra of FLV-UK-1 (A), FLV-UK-2 (**B**), and FLV-UK-3 (**C**) detected in the positive ion mode. The retention times of FLV-UK-1, FLV-UK-2, and FLV-UK-3 were 10.8, 6.4, and 6.6 min, respectively. The FLV-UK-1 peak in Fig. 2C, the FLV-UK-2 peak in Fig. 2F, and the FLV-UK-3 peak in Fig. 2I were subjected to further LC-Q-Orbitrap-MS analysis (PRM, MSMS analyses). The assignment of FLV-UK-1, FLV-UK-2, and FLV-UK-3 precursor and product ions is summarized in Fig. 3A and C and Tables S1–S3

The results of MS/MS analyses of FLV-UK-1 are shown in Fig. 3A. One precursor ion (FLV-UK-1-Fr.1) and five product ions (FLV-UK-1-Fr.2–FLV-UK-1-Fr.6) with *m/z* values of 347.1577, 258.1100, 226.0839, 200.0683, 87.0809, and 71.0497, respectively, were detected. Notably, Δ*m/z* values for the precursor and product ions of FLV-UK-1 were < ± 10 ppm (Table S2). The assignment of the precursor and product ions of FLV-UK-1 is shown in Fig. 3A and Table S2. FLV-UK-1 was designated (*E*)-*N*-(2-(((5-methoxy-1-(4(trifluoromethyl)phenyl)pentylidene)amino)oxy)ethyl)formamide.

The results of MS/MS analyses of FLV-UK-2 are shown in Fig. 3B. One precursor ion (FLV-UK-2-Fr.1) and four product ions (FLV-UK-2-Fr.2–FLV-UK-2-Fr.5) with *m/z* values of 305.1456, 244.0940, 229.0835, 200.0674, and 77.0715, respectively, were detected. Notably, Δ*m/z* values for the precursor and product ions of FLV-UK-2 were < ± 10 ppm (Table S3). The assignment of the precursor and product ions of FLV-UK-2 is shown in Fig. 3B and Table S3. FLV-UK-2 was designated (*E*)-5-hydroxy-1-(4-(trifluoromethyl)phenyl)pentan-1-one *O*-(2-aminoethyl) oxime.

The results of MS/MS analyses of FLV-UK-3 are shown in Fig. 3C. One precursor ion (FLV-UK-3-Fr.1) and four product ions (FLV-UK-3-Fr.2–FLV-UK-3-Fr.5) with *m/z* values of 335.1564, 317.1462, 256.0941, 103.0756, and 71.0497, respectively, were detected. Notably, Δ*m/z* values for the precursor and product ions of FLV-UK-3 were < ± 10 ppm (Table S4). The assignment of the precursor and product ions of FLV-UK-3 is shown in Fig. 3C and Table S4. FLV-UK-3 was designated (*E*)-5-(hydroxymethoxy)-1-(4-(trifluoromethyl)phenyl)pentan-1-one *O*-(2-aminoethyl) oxime.

**Fig. 4:**
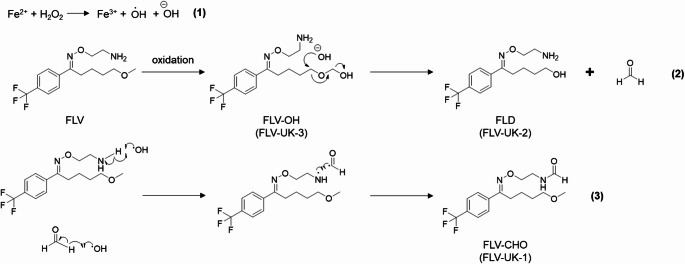
Schematic diagram of FLV degradation in the reaction mixture containing FeCl_2_/H_2_O_2_

### Changes in FLV concentration and detection of FLV degradation products in human blood

FLV concentration in human blood maintained at 37 °C showed a significant decrease at 168 h compared with that at 0 h (Fig. S1A). The percentages of undegraded FLV in human blood at 24, 96, and 168 h relative to those at 0 h were 95.0 ± 2.8%, 56.9 ± 3.6%, and 54.9 ± 2.6%, respectively.

The peak area of FLV-UK-1 in human blood maintained at 37 °C was higher at 168 h than at 0 h (Fig. S1B). The peak areas of FLV-UK-1 in human blood at 0, 24, 96, and 168 h were 857 × 10^2^ ± 470 × 10 a.u., 840 × 10^3^ ± 270 × 10^2^ a.u., 158 × 10^4^ ± 110 × 10^3^ a.u., and 188 × 10^4^ ± 672 × 10^2^ a.u., respectively.

**Fig. 5:**
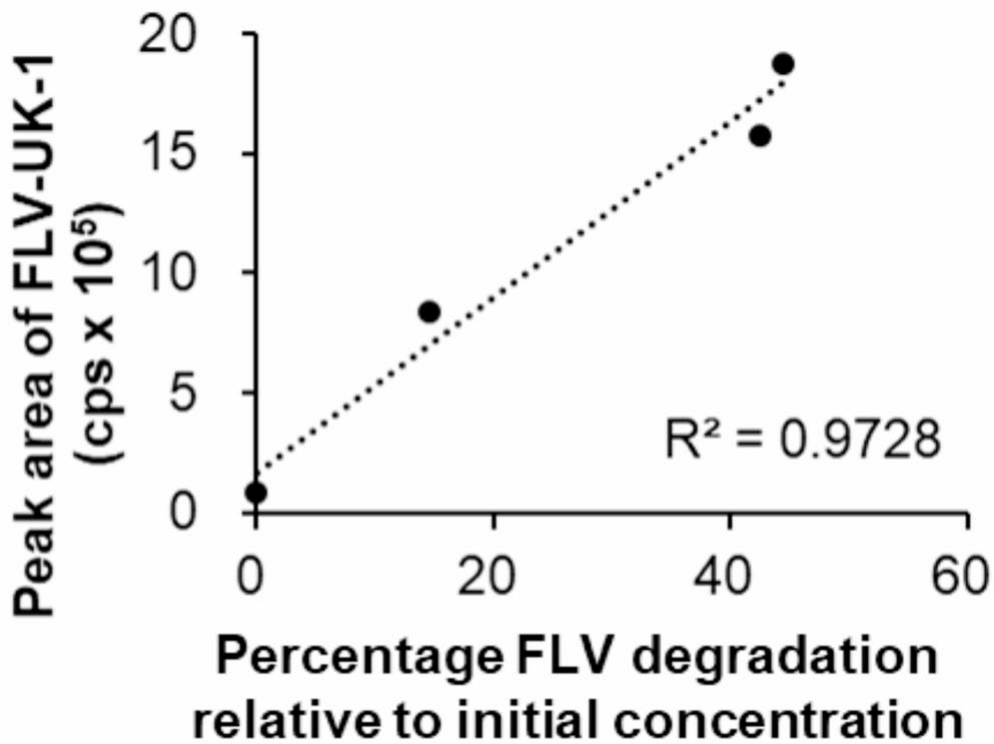
Relationship between peak area of FLV-UK-1 and percentage FLV degradation relative to its initial concentration on in vitro study using human blood

The peak area of FLV-UK-1 was plotted against percentage FLV degradation relative to its initial concentration Fig. 4, Fig. 5). A strong positive correlation was noted between the peak area of FLV-UK-1 and the percentage FLV degradation relative to its initial concentration, with a correlation coefficient (r^2^) of 0.97.

FLV-UK-2 and FLV-UK-3 were not detected in human blood for 168 h.

## Discussion

We found that FLV was degraded by hydroxide ions and hydroxyl radicals generated by Hb/H_2_O_2_ and FeCl_2_/H_2_O_2_ (Figs. 1 and 5), as expected. It has been reported that Hb seems to degrade several drugs in PM blood [7–13]. Moreover, FLV is the target of the formation of adducts with formaldehyde produced from FLV (Figs. 3 and 5). Our findings indicate a new mechanism for the change in the free form of FLV by Hb/H_2_O_2_ in human blood.

As shown in the schematic diagram in Fig. 4, FLV-CHO, FLD, and FLV-OH were detected in the FeCl_2_/H_2_O_2_ reaction mixture. In contrast, only FLV-CHO was detected in the Hb reaction mixture and human blood (Figs. 2 and 5 and S2). It has been reported that reactants, such as methomyl and malathion, bind to the residue of blood proteins, such as Hb [12, 13]. These results suggest that because FLV-OH might bind to Hb residue (Fig. S3), FLV-OH and FLD produced from it would not be detected in Hb solution and human blood. Thus, only FLV-CHO is useful in forensic toxicology.

In forensic toxicology, blood drug concentration is critical for determining drug intoxication. FLV concentration in blood decreases over time [5]. To prove FLV intoxication, a biomarker in blood to estimate FLV concentration at the time of death is required. In this study, we detected FLV-CHO in human blood at each time point and found that its concentration is strongly correlated with percentage FLV degradation relative to its initial concentration (Fig. 5). These results suggest that FLV-CHO may be a valuable biomarker for estimating FLV concentration at the time of death. In addition, eleven metabolites, including FLD produced by human liver microsomes, were detected. To our knowledge, FLV-CHO is not detected in the blood of live individuals, as it is formed solely by Hb/H_2_O_2_. In this regard, FLV-CHO can be an indicator of substantial postmortem degradation of FLV.

## Conclusion

FLV is degraded in human blood, and degradation products FLV-CHO, FLD, and FLV-OH are generated via oxidation and/or hydroxylation of the free form of FLV through the Fenton reaction with Hb. FLV-CHO in blood can serve as a valuable biomarker for estimating FLV concentration at the time of death. In addition, FLV-CHO can be an indicator of substantial postmortem degradation of FLV.

## Supplementary Information

Below is the link to the electronic supplementary material.

Supplementary Material 1
