# Supplementary material for: Characterization of fluvoxamine degradation products in postmortem blood by liquid chromatography coupled with quadrupole-Orbitrap mass spectrometry
Source: Forensic Toxicol. 2026 Mar 16;44(2):349–55. doi: 10.1007/s11419-026-00763-6 (PMC13303306; doi:10.1007/s11419-026-00763-6)
Supplement: Supplementary file 1 — Supplementary Material 1 [file 11419_2026_763_MOESM1_ESM.docx]

**Supporting Information**

**Characterization of fluvoxamine degradation products in blood by liquid chromatography coupled with quadrupole-Orbitrap mass spectrometry**

Yoshikazu Yamagishi^a*^, Kazuaki Takahashi^b^, Hiroyuki Inoue^a^, Sayaka Nagasawa^a,c^,

Hirotaro Iwase^a^, and Yasumitsu Ogra^a,c,d^

^a^*Department of Legal Medicine, Graduate School of Medicine, Chiba University, 1-8-1 Inohana, Chuo, Chiba 260-8670, Japan*

^b^*Graduate School of Horticulture, Chiba University, 1-33 Yayoi-cho, Inage, Chiba 263-8522, Japan*

*^c^Laboratory of Forensic Toxicology, Graduate School of Pharmaceutical Sciences, Chiba University, 1-8-1 Inohana, Chuo, Chiba 260-8675, Japan*

^d^*Laboratory of Toxicology and Environmental Health, Graduate School of Pharmaceutical Sciences, Chiba University, 1-8-1 Inohana, Chuo, Chiba 260-8675, Japan*

***Corresponding Author**

Yoshikazu Yamagishi, Ph.D.

Department of Legal Medicine, Graduate School of Medicine, Chiba University

Tel: 81-43-226-2078 Fax: 81-43-226-2079

E-mail: yamagishiyo@chiba-u.jp

**Table of contents**

**Table S1** Instrumentation and operational settings for liquid chromatography and mass spectrometry

**Table S2** Assignment of FLV-UK-1 precursor and product ions

**Table S3** Assignment of FLV-UK-2 precursor and product ions

**Table S4** Assignment of FLV-UK-3 precursor and product ions

**Figure S1** Time course of FLV concentration (A) and peak area of FLV-UK-1 in human blood (B).

One µg/mL FLV was incubated in human blood for 168 hr. The temperature was kept at 37ºC. Dunnett’s multiple comparison test was used for comparisons among four groups. Double asterisks (**) indicate levels of significance at *p* < 0.01 (*n* = 4).

**Figure S2**  ^1^H NMR data (400 MHz, CDCl_3_) of FLV-CHO purified from the reaction mixture by the Hb/H_2_O_2_ reaction mixture.

**Figure S3** Proposed pathway for FLV degradation in the Hb/H_2_O_2_ reaction mixture.

| **Table S1. Instrumentation and operational settings for liquid chromatography and mass spectrometry** | |
| --- | --- |
| Liquid chromatography | Vanquish Flex Binary LC |
| Column | CORTECS T3 column, 100 × 2.1 mm I.D., 2.7 μm, (Waters, Milford, MA, USA) |
| Oven [ºC] | 40 |
| Total flow [mL/min] | 0.3 |
| Elution buffer A | 0.1% Formic acid and 0.01 M ammonium formate |
| Elution buffer B | Acetonitrile |
| Gradient curve [A/B] | 90/10 (0 min)–40/60 (12 min)–0/100 (18–23 min)–  90/10 (23–30 min) |
| Injection volume [µL] | 1 (Full-scan MS) or 10 (parallel reaction monitoring, PRM) |
|  |  |
| Mass spectrometry | Q-Exactive Plus Orbitrap mass spectrometer |
| Polarity | Positive |
| Mode | Full-scan MS and PRM |
| *m/z* range | 100–500 |
| Heater temperature [°C] | 413 |
| Capillary temperature [°C] | 256 |
| Auxiliary gas [a.u.] | 11 |
| Sheath gas [a.u.] | 48 |
| S-lens radio frequency level | 50 |
| Spray voltage [kV] | 3.5 |
| AGC target value | 5 e^6^ (Full-scan MS), 1 e^5^ (PRM) |
| Resolution at *m/z* 200 | 70,000 (Full-scan MS), 17,500 (PRM) |
| Collision energy [V] | 10, 20, and 30 (FLV-UK-1), 10 (FLV-UK-2 and FLV-UK-3) |

| **Table S2. Assignment of FLV-UK-1 precursor and product ions** | | | |  |
| --- | --- | --- | --- | --- |
| Peak no. | Elemental  composition | Theoretical  *m/z* | Measured  *m/z* | Δ *m/z*  [ppm] |
| FLV-UK-1-Fr.1 | C_16_H_22_F_3_N_2_O_3_ | 347.1577 | 347.1577 | 0.0 |
| FLV-UK-1-Fr.2 | C_13_H_15_F_3_NO | 258.1100 | 258.1100 | 0.0 |
| FLV-UK-1-Fr.3 | C_12_H_11_F_3_N | 226.0838 | 226.0839 | 0.4 |
| FLV-UK-1-Fr.4 | C_10_H_9_F_3_N | 200.0682 | 200.0683 | 0.5 |
| FLV-UK-1-Fr.5 | C_5_H_11_O | 87.0804 | 87.0809 | 5.7 |
| FLV-UK-1-Fr.6 | C_4_H_7_O | 71.0491 | 71.0497 | 8.4 |

| **Table S3. Assignment of FLV-UK-2 precursor and product ions** | | | |  |
| --- | --- | --- | --- | --- |
| Peak no. | Elemental  composition | Theoretical  *m/z* | Measured  *m/z* | Δ *m/z*  [ppm] |
| FLV-UK-2-Fr.1 | C_14_H_20_F_3_N_2_O_2_ | 305.1471 | 305.1458 | -4.3 |
| FLV-UK-2-Fr.2 | C_12_H_13_F_3_NO | 244.0944 | 244.0940 | -1.6 |
| FLV-UK-2-Fr.3 | C_12_H_12_F_3_O | 229.0835 | 229.0835 | 0.0 |
| FLV-UK-2-Fr.4 | C_10_H_9_F_3_N | 200.0682 | 200.0674 | -4.0 |
| FLV-UK-2-Fr.5 | C_2_H_9_N_2_O | 77.0709 | 77.0715 | 7.8 |

| **Table S4. Assignment of FLV-UK-3 precursor and product ions** | | | |  |
| --- | --- | --- | --- | --- |
| Peak no. | Elemental  composition | Theoretical  *m/z* | Measured  *m/z* | Δ *m/z*  [ppm] |
| FLV-UK-3-Fr.1 | C_15_H_22_F_3_N_2_O_3_ | 335.1564 | 335.1564 | 0.0 |
| FLV-UK-3-Fr.2 | C_15_H_20_F_3_N_2_O_2_ | 317.1471 | 317.1462 | -2.8 |
| FLV-UK-3-Fr.3 | C_13_H_13_F_3_NO | 256.0944 | 256.0941 | -1.2 |
| FLV-UK-3-Fr.4 | C_5_H_11_O_2_ | 103.0754 | 103.0756 | 1.9 |
| FLV-UK-3-Fr.5 | C_4_H_7_O | 71.0491 | 71.0497 | 8.4 |


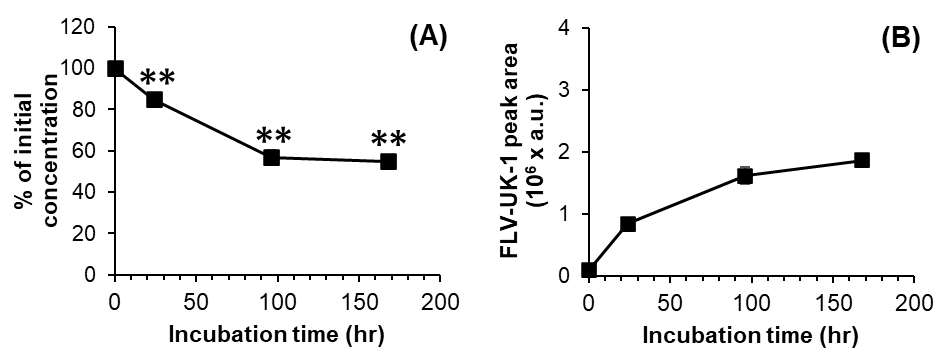


**Figure S1** Time course of FLV concentration (A) and peak area of FLV-UK-1 in human blood (B).

One µg/mL fluvoxamine was incubated in human blood for 168 hr. The temperature was maintained at 37ºC. Dunnett’s multiple comparison test was performed for comparisons among four groups. Double asterisks (**) indicate levels of significance at *p* < 0.01 (*n* = 4).


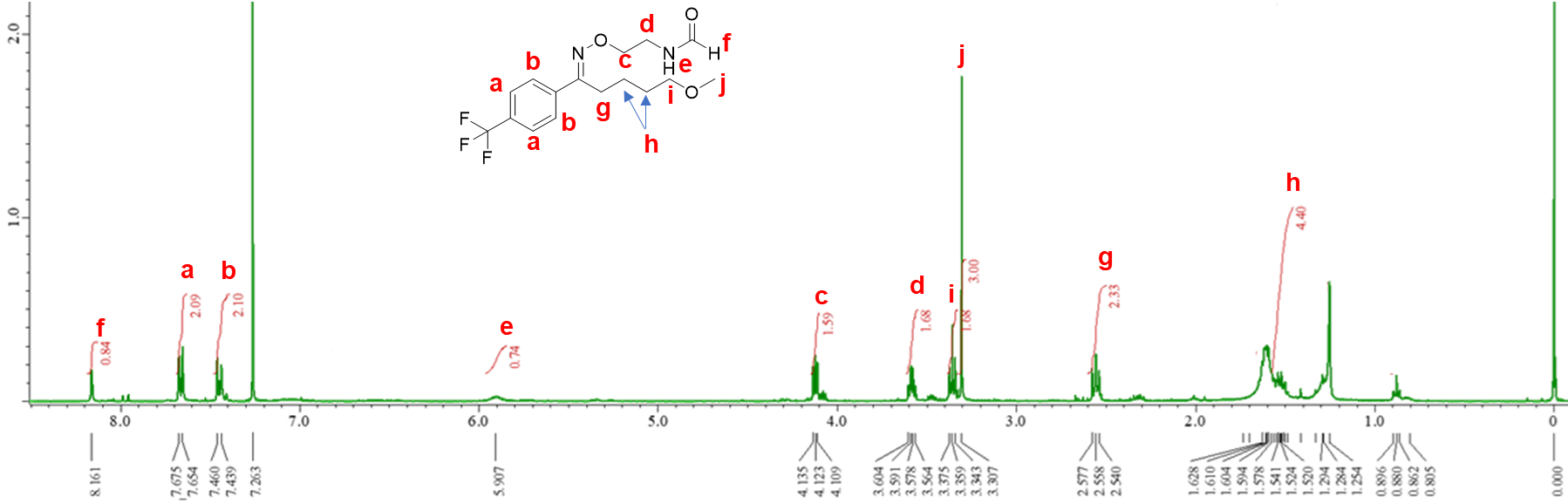


**Figure S2** ^1^H NMR data (400 MHz, CDCl_3_) of FLV-CHO purified from the reaction mixture by the Hb/H_2_O_2_ reaction mixture.


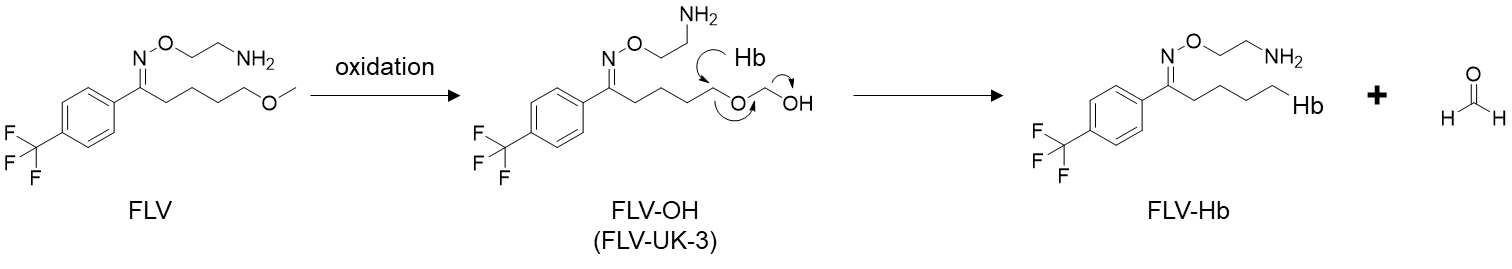


**Figure S3** Proposed pathway for FLV degradation in the Hb/H_2_O_2_ reaction mixture.
